# Binding interaction of a novel fluorophore with serum albumins: steady state fluorescence perturbation and molecular modeling analysis

**DOI:** 10.1186/s40064-015-1333-8

**Published:** 2015-09-24

**Authors:** Uttam Pal, Sumit Kumar Pramanik, Baisali Bhattacharya, Biswadip Banerji, Nakul Chandra Maiti

**Affiliations:** Structural Biology and Bioinformatics Division, Council of Scientific and Industrial Research (CSIR)-Indian Institute of Chemical Biology (IICB), Kolkata, West Bengal India; Chemistry Division, Council of Scientific and Industrial Research (CSIR)-Indian Institute of Chemical Biology (IICB), Kolkata, West Bengal India

**Keywords:** Serum albumin, New chemical entity, Binding site, Microenvironment, Fluorescence probe

## Abstract

Fluorescence emission and anisotropy are widely used to measure the binding parameters and kinetic behavior of reactions that cause a change in the rotational time of a fluorescent molecule. We report here fluorescence emission and anisotropy behavior of a newly synthesized novel naphthalene base fluorophore (methyl 3-[(6-{[2-(tert-butoxy)-2-oxoethyl] (4-methoxyphenyl)amino}naphthalen-2-yl)formamido]propanoate) in several solution conditions including its binding to human and bovine serum albumin proteins both in their native and denatured states. The fluorescence yield of the compound substantially increased inside hydrophobic protein surface and ~30 nm decrease in Stokes’ shift, compared to aqueous solution, was observed. Shift in fluorescence excitation peak position from the absorption peak of the molecule was ~8 nm in protein solution. This indicated possible alteration of excited state geometry of the compound by the globular fold of albumins. In addition, we measured the steady state fluorescence anisotropy of the molecule to evaluate several thermodynamic parameters and the results suggested the binding was energetically favorable. The measured ΔG° was ~−30 kJ mol^−1^ and the derived dissociation constant was ~10^−6^ M. The molecular docking analysis further highlighted the nonspecific association of the compound with the proteins and hydrophobic forces may have a significant role in the binding processes. Under the denatured condition of the protein, the compound lost its binding efficacy and reduction in fluorescence intensity was observed. Thus, the molecule appears as a new fluorescence probe to report the nature of its binding site in terms of increased fluorescence quantum yield and decreased Stokes’ shift. It can also report the changes in the binding site due to global change in protein structure such as unfolding/misfolding often linked to several human disorder. Further it could be useful to detect and study the drug binding site of specific protein of interest.

**Electronic supplementary material:**

The online version of this article (doi:10.1186/s40064-015-1333-8) contains supplementary material, which is available to authorized users.

## Background

The interaction and the energetics of small molecule binding to a protein largely depend on the molecular architecture and microenvironment provided due to folding/unfolding or even transformation of the protein structure. The observable properties of a small molecule in such microenvironment, in turn, carry information about the binding site, which is crucial for drug development (Royer 2006; Cohen et al. 2002; Abou-Zied and Al-Shini 2008; Er et al. 2013) and many other investigations. Our attention was focused on fluorescence emission and binding aspects of the fluorophore in the hydrophobic milieu inside a globular fold of a protein under physiological and denaturing conditions. We have synthesized a naphthalene based fluorophore, methyl 3-[(6-{[2-(tert-butoxy)-2-oxoethyl](4-methoxyphenyl)amino}naphthalen-2-yl) formamido]propanoate (compound **5**) to study the immediate surroundings of the molecule inside the proteins.

Serum albumin was chosen as model protein with at least seven hydrophobic grooves on its surface. It provides a unique microenvironment and acts as a universal receptor for many drug molecules (Er et al. 2013; Reichenwallner and Hinderberger 2013; Simard et al. 2006; Curry et al. 1998). This protein increases the solubility of hydrophobic ligands in plasma and modulates their delivery to cells. The precise architecture of the binding pockets is known from several crystallographic and NMR spectroscopic studies (Reichenwallner and Hinderberger 2013; Simard et al. 2006; Curry et al. 1998). Thus, the interaction pattern and the spectroscopic signature of a small molecule housed in the well defined environment of serum albumin could provide significant insight into the interaction pattern and its binding efficacy (Yamasaki et al. 2013).

Intrinsic protein fluorescence originating from tryptophan and tyrosine residues or the fluorescence of the drug molecule itself provides ample information about the local environment, the changes in protein conformation and the interaction of a protein with a drug molecule (Royer 2006; Cohen et al. 2002; Abou-Zied and Al-Shini 2008). However, the current investigation explored both the quantitative and qualitative aspect of the interaction and incorporation of compound **5** into the binding pockets of serum albumin, at the molecular level using fluorescence methodologies. The thermodynamic parameters were obtained by measuring the effect of temperature on binding constant. In addition to access the interaction site and binding specificity of the drug molecule computational modeling analysis was carried out.

## Methods

### Chemicals

Bovine and human serum albumins were purchased from Sigma–Aldrich Corporation (St. Louis, MO, USA). Tris–HCl and Urea were also purchased from Sigma-Aldrich. All the samples were prepared in 20 mM Tris–HCl buffer of pH 7.0. Deionized and triple distilled water was used for preparing buffer solution that was passed through 0.22 µm pore size Millipore filters (Millipore India Pvt. Ltd., Bangalore, India).

All air and water sensitive reactions were carried out in oven dried glassware under nitrogen atmosphere using standard manifold techniques. All the chemicals were purchased from Acros organics and Sigma-Aldrich, and used without further purification unless otherwise stated. Compounds that are not described in the experimental part were synthesized according to the literature procedures. Solvents were freshly distilled by standard procedures prior to use. Flash chromatography was performed on silica gel (Merck, 100–200 mesh) with the indicated elutant. All ^1^H and ^13^C-NMR spectra were recorded on a Bruker 600 MHz spectrometer. For ^1^H NMR, tetramethylsilane (TMS) served as internal standard (δ = 0) and data are reported as follows: chemical shift, integration, multiplicity (s = singlet, d = doublet, t = triplet, q = quartet, m = multiplet) and coupling constant(s) in Hz. For ^13^C NMR, TMS (δ = 0) or CDCl_3_ (δ = 77.26) was used as internal standard and spectra were obtained with complete proton decoupling. Mass spectra were obtained on a Jeol MS station 700 and ESI-TOF mass spectrometer.

### Procedure to synthesize compound **5**

**Scheme 1:**
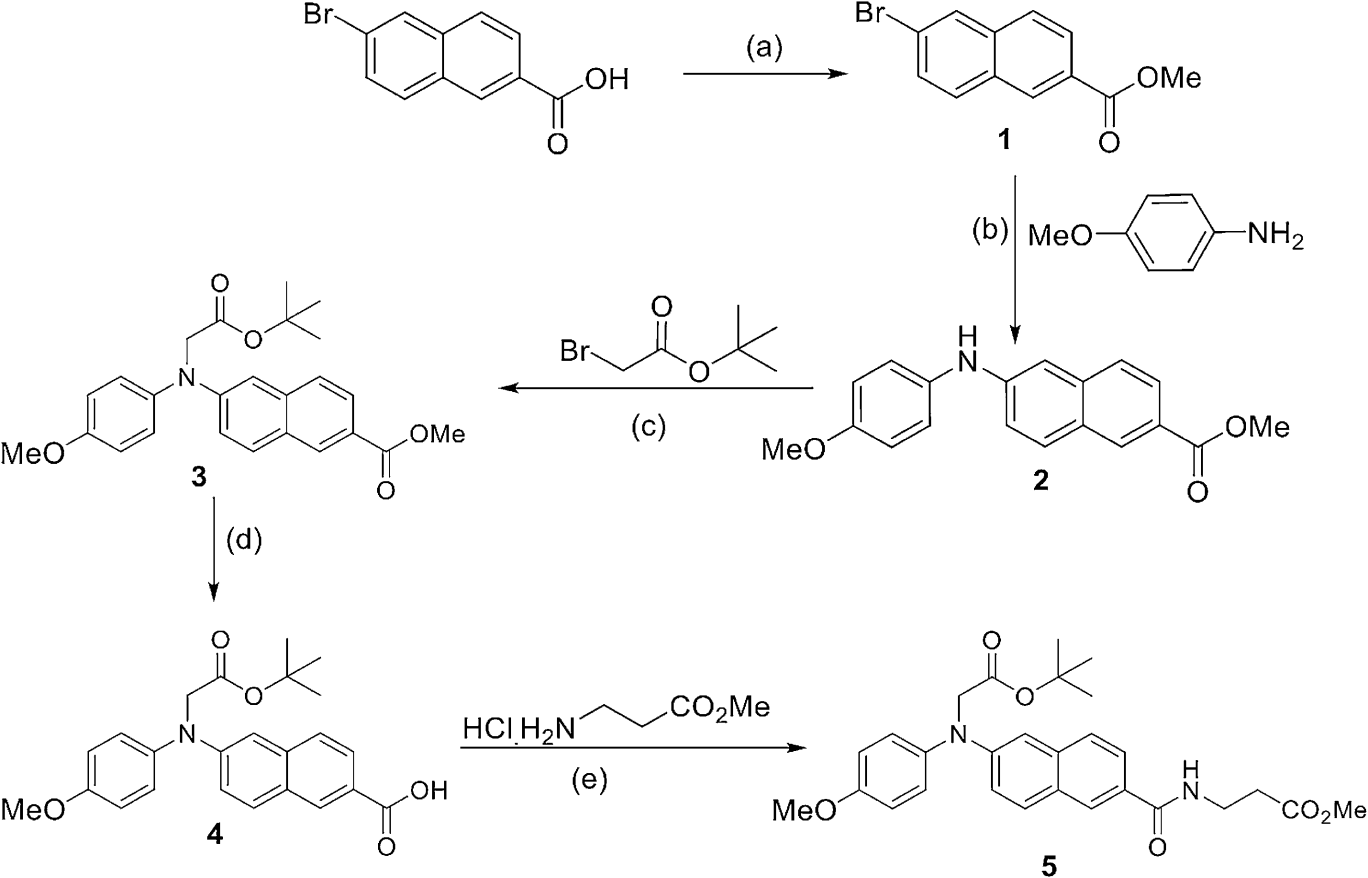
Reagent and conditions: *a* Conc. H_2_SO_4_, MeOH, 0 °C to r.t., 6 h. *b* Palladium (II) acetate (0.05 equiv.), xantphos (0.1 equiv.) and cesium carbonate (3 equiv.), 80 °C, 4 h. *c* Potassium tert-butoxide (1.2 equiv.), DMF, 0 °C to r.t., 12 h. *d* Lithium hydroxide (3 equiv.), MeOH–water (5:1), r.t., 2 h. *e* EDC.HCl (3.0 equiv.), HOBT (2.5 equiv.), TEA (6 equiv.), 0 °C to r.t., 1.5 h

Synthesis was carried out following the Scheme 1. Detailed procedure and characteristic data are given in the Additional file 1.

### Absorption spectroscopy

Ground-state absorption spectra were recorded with a Shimadzu UV-2401PC Spectrometer. 1 cm path-length quartz cuvette was used and 250–450 nm wavelength range was scanned. Compound **5** absorption spectra as a function of BSA and HSA concentration were recorded by titrating (Banerjee et al. 2012; Ray et al. 2012) compound **5** solution with concentrated protein solutions. Small dilution error in the titration experiment was ignored.

### Fluorescence emission and excitation spectroscopy

The steady-state fluorescence emission and excitation spectra were recorded with a Cary Eclipse Fluorescence Spectrophotometer. The emission spectra of serum albumins and compound **5** were obtained by exciting the samples at the wavelengths 295 and 330 nm, respectively. The excitation spectra of compound **5** was obtained by recording the emission at wavelength 450 nm. In all the cases, the excitation and emission slit widths were kept at 5 nm each. Compound **5** fluorescence emission or excitation spectra as a function of protein concentration were recorded by simple titration method (Banerjee et al. 2012, 2013; Ray et al. 2012).

### Fluorescence anisotropy

Fluorescence anisotropy experiments were performed in the Cary Eclipse Fluorescence Spectrophotometer and a manual polarizer accessory was used. The excitation and emission wavelengths were set to 330 and 450 nm, respectively, with slit widths of 5 nm for each monochromator. Anisotropy (r) was determined using the following equation (Banerjee et al. 2012):

1 $${\text{r}} = \left( {{\text{I}}_{\text{VV}} {-}{\text{G}}*{\text{I}}_{\text{VH}} } \right)/\left( {{\text{I}}_{\text{VV}} + 2 {\text{G}}*{\text{I}}_{\text{VH}} } \right);{\text{ G}} = {\text{I}}_{\text{HV}} /{\text{I}}_{\text{HH}}$$

where I is the fluorescence emission intensity when excitation and emission polarizes are aligned in a particular way denoted by the suffix. For example, VV indicates both the excitation and emission polarizers are vertically aligned whereas VH indicates excitation polarizer is vertical and the emission polarizer is horizontally aligned and so on. G is a correction factor.

The changes in compound **5** fluorescence anisotropy as a function of protein concentration were recorded by simple titration method as mentioned earlier.

### Determination of binding constants

K_d_ for compound **5** binding with BSA and HSA were determined from the compound **5** fluorescence anisotropy perturbation with proteins. Compound **5** concentration was kept at 0.5 µM and the protein concentration was varied from 0 to 5.5 µM. Small dilution error due to the titration was ignored. Anisotropy values as a function of protein concentration were recorded. To derive the binding parameters, data were analyzed using the non-linear Langmuir isotherm (Banerji et al. 2013):

2 $$\Delta {\text{r}} = \Delta {\text{r}}_{ \text{max} } *\left[ {\text{P}} \right]/\left( {{\text{K}}_{\text{d}} + \left[ {\text{P}} \right]} \right)$$

where Δr is the difference in fluorescence anisotropy in the absence and presence of the protein at concentration [P], Δr_max_ is the maximum possible change in the fluorescence anisotropy, *K*_*d*_ is the binding dissociation constant. The non-linear equation was fitted to the data using Wolfram Mathematica 10.

Stern–Volmer quenching constant or the binding affinity constant, K_a_ was determined as a reciprocal of K_d_ (Banerji et al. 2013).

### Binding thermodynamics

*K*_*d*_ values were determined as a function of temperature and the thermodynamic parameters of binding was obtained by fitting van’t Hoff equation (Banerjee et al. 2012; Ray et al. 2012) to the data:

3 $${\text{ln K}}_{\text{eq}} = - \Delta {\text{H}}^\circ /{\text{RT}} + \Delta {\text{S}}^\circ /{\text{R}}$$

where K_eq_ is the equilibrium constant (here the Stern–Volmer quenching constant) of binding at corresponding temperature T, and R is the gas constant. The equation gives the standard enthalpy change (ΔH°) and standard entropy change (ΔS°) on binding. The free energy change (ΔG°) has been estimated from the following relationship (Banerjee et al. 2012; Ray et al. 2012):

4 $$\Delta {\text{G}}^\circ = \Delta {\text{H}}^\circ - {\text{T}}\Delta {\text{S}}^\circ$$

### Thermal and chemical denaturation

Thermal denaturation of protein was performed by increasing the temperature of the compound **5**-protein solution from 10 to 80 °C in 8 steps (Banerjee et al. 2012). The sample was kept under continuous stirring condition and the changes in compound **5** fluorescence spectra were recorded. Chemical denaturation was achieved by increasing the concentration of urea (Banerjee et al. 2012) while recording the compound **5** fluorescence.

### Lipophilicity and solubility calculations

Lipophilicity in terms of calculated (clogP) and solubility in terms of calculated logS (clogS) were determined at Virtual Computational Chemistry Laboratory server (http://www.vcclab.org/lab/alogps/) (Tetko et al. 2005). Polar surface area was calculated with a 1.4 Å radius probe size.

### Molecular docking

Molecular docking experiments were performed using AutoDock 4.2 (Morris et al. 2009) and AutoDock Vina (Trott and Olson 2010) of The Scripps Research Institute and the SwissDock server (http://www.swissdock.ch/). AutoDock 4.2, Vina and SwissDock uses different algorithm and scoring function for docking calculations. AutoDockTools (Morris et al. 2009) was used for the preparation of ligands and proteins for docking. BSA (PDB: 3V03) (Majorek et al. 2012) and HSA (PDB: 1E78) (Bhattacharya et al. 2000) structural information were obtained from Protein Data Bank (Berman et al. 2000). The ligand structures were drawn in Avogadro (Hanwell et al. 2012) and geometry optimized in vaccuo using the steepest descent followed by conjugate gradient algorithms. Genetic algorithm was used in AutoDock 4.2 and it was run (ga_run) 100 times to generate a statistically significant number of docked poses (Alam et al. 2012). AutoDock 4.2 results were clustered using binding free energy and standard deviation cut offs of 0.5 kcal mol^−1^ and 2Å, respectively.

## Results and discussion

### Absorbance and fluorescence of compound **5**

**Fig. 1:**
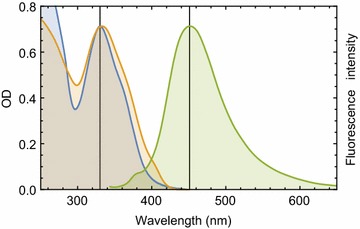
Absorption and fluorescence behavior of compound **5** in the UV–Visible range. Absorption spectrum of compound **5** in 20 mM Tris–HCl buffer of pH 7.4 is shown in* blue*. Fluorescence emission and excitation spectra of compound **5** are shown in *green* and *yellow*, respectively. Compound **5** fluorescence spectra were recorded in the same buffer and normalized against its absorption spectrum

In aqueous buffer at pH 7.4, Compound **5** shows a strong absorption band with a peak at 330 nm. Compound **5** is also fluorescence active and the fluorescence band appeared at ~450 nm (Fig. 1). Figure 1 also shows the excitation spectrum of compound **5**, which largely overlapped with the absorption spectrum suggesting that excited state conformation of compound **5** in solution is homogeneous and close to ground state structure.

### Fluorescence quantum yield of compound **5** in protein environment

**Fig. 2:**
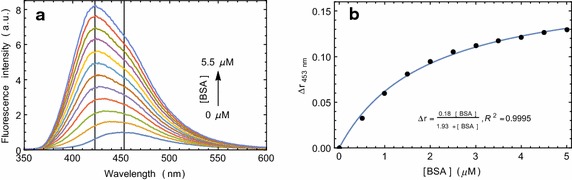
Fluorescence emission and anisotropy change of compound **5** in presence of serum albumin. Compound **5** concentration was kept constant at 0.5 μM and the protein concentration was varied from 0 through 5.5 μM. **a** The changes in fluorescence emission spectrum of compound **5** as a function of BSA concentration. **b** The changes in compound **5** fluorescence anisotropy with increasing concentration of BSA and the fitted Langmuir isotherm

Compound **5** absorbs UV–visible light strongly at the wavelengths where protein absorbs. It also has a strong absorption band in the fluorescence emission range of protein. Therefore, protein intrinsic fluorescence perturbation with compound **5** or the energy transfer from protein to compound **5** is not an eminent choice to probe the binding interactions of compound **5** with proteins. However, the changes in compound **5** fluorescence may be monitored as a parameter of binding. With the increasing concentration of serum albumins, we have found that the fluorescence intensity of the compound **5** increases (Fig. 2a, Additional file 1: Figure S1A). A blue shift in the emission maximum was also observed. It indicated compound **5** binding to the hydrophobic grooves of serum albumins. Such binding causes solvent exclusion of compound **5** and the energy that is otherwise spent in solvent relaxation, is gained by the emitting photons. Here, about 30 nm decrease in the Stokes’ shift in compound **5** fluorescence corresponds to ~0.2 eV energy gain by each emitted photon.

Interaction of compound **5** with serum albumins increases its fluorescence quantum yield and compound **5** gets a hydrophobic environments while bound to the serum albumins. Thus, the changes in compound **5** fluorescence in the presence of proteins carry the information not only about the interaction but the microenvironment of the binding site on its target protein as well.

### Fluorescence anisotropy of compound **5** in presence of proteins

Small molecules tumble faster in less viscous solvents. But when it binds to a large molecule such as protein, its movement gets restricted. Fluorescence anisotropy is, therefore, widely used to measure the binding constants and kinetics of reactions that cause a change in the rotational time of the fluorescent molecules (Heyduk et al. 1996). Fluorescence anisotropy measurements can also elucidate the microenvironment of a small molecule in terms of its rotational diffusion, interactions, and proximity to proteins. Compound **5** in buffer solution shows very low anisotropy. With the increasing concentration of serum albumins, fluorescence anisotropy of compound **5** increases and gradually reaches the saturation (Fig. 2b, Additional file 1: Figure S1B). Binding dissociation constants for compound **5** binding with the two serum albumins were determined from this experiment and were found to be in the low micromolar concentration range (Table 1). The molecule showed increased quantum yield along with a blue shift in presence of protein. Anisotropy experiment confirmed that the molecule goes inside the binding cavity of the protein, thus, restricting its free rotation. Therefore, the anisotropy suggests that the observed change in the fluorescence property of the molecule is a direct effect of the binding site environment.

**Table 1:** The K_d_ and K_a_ values for the binding of the compound **5** to serum albumins as determined by the fluorescence and anisotropy perturbation experiments at room temperature

Protein	K_d_ (M)	K_a_ (M^−1^)
BSA	1.93 × 10^−6^	5.18 × 10^5^
HSA	2.05 × 10^−6^	4.88 × 10^5^

### Thermodynamics of compound **5** binding to serum albumins

**Fig. 3:**
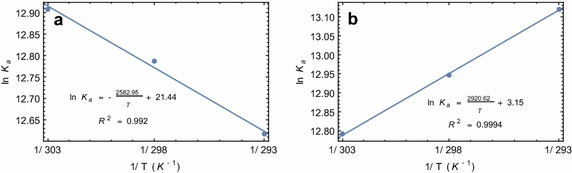
Determination of thermodynamic parameters of compound **5** binding from van’t Hoff’s plot. **a** Decrease in the binding equilibrium constant with the decreasing temperature for BSA-compound **5** interaction and the fitted van’t Hoff equation. **b** Increase in the binding equilibrium constant with the decreasing temperature for HSA-compound **5** interaction and the fitted van’t Hoff equation

Equilibrium constant of a reaction changes with the temperature (Fig. 3). Such a change can be explained by van’t Hoff’s equation, which in turn, gives the standard enthalpy and standard entropy changes for the reaction. The associations of the compound with serum albumins are thermodynamically favorable, which is evident from the decrease in Gibbs free energy (Table 2). Moreover, the binding with HSA is enthalpy driven (negative ΔH°) whereas the binding with BSA is entropy driven (positive ΔS°). It suggests that, despite the structural similarity in the two proteins, the interactions with HSA are thermodynamically different from that of BSA.

**Table 2:** Thermodynamics of compound **5** binding to serum albumins

Serum albumins	ΔG° (kJ mol^−1^) at 25 °C	ΔH° (kJ mol^−1^)	ΔS° (J mol^−1^ K^−1^)
BSA	−31.64	21.47	178.25
HSA	−32.09	−24.28	26.18

### Excited state geometry of compound **5** in protein environment

**Fig. 4:**
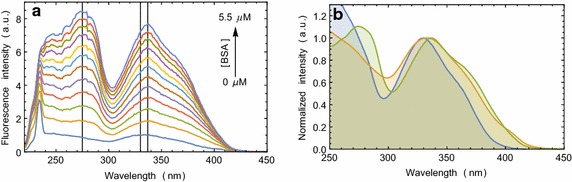
Compound **5** binding is an excited state phenomenon. **a** The changes in compound **5** fluorescence excitation spectra upon addition of BSA. **b** Normalized absorption (*blue*) and fluorescence excitation (*yellow*) spectra of compound **5** overlapped with fluorescence excitation spectrum (*green*) in presence of BSA

Fluorophore binding to a protein often results in an altered ground state electronic property, which can be visualized by a change in the absorption spectrum. However, the absorption spectrum of compound **5** does not change due to its binding with the serum albumins (Additional file 1: Figure S2). It indicates that the ground state geometry of compound **5** inside the hydrophobic groove of serum albumins remains the same as in the solution. Interestingly, when we recorded the excitation spectrum of compound **5** in presence of serum albumins (Fig. 4, Additional file 1: Figure S3), we observed ~8 nm red shift in the wavelength of excitation maximum (330–338 nm). It indicates that the excited state geometry of compound **5** within the binding site of serum albumin gets altered with the possible formation of an exciplex.

### Denaturation of proteins bound to compound **5**

**Fig. 5:**
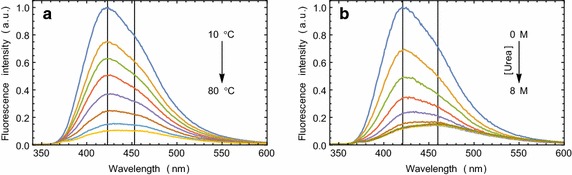
Compound **5** interaction with denaturing proteins. **a** The changes in fluorescence of BSA compound **5** complex with the increasing temperature. **b** The changes in fluorescence of BSA compound **5** complex with the increasing concentration of Urea

Serum albumin-compound **5** complexes were denatured chemically and thermally. When compound **5** is bound to the protein, fluorescence is blue shifted. With the gradual increase in the temperature or the urea concentration, the fluorescence intensity of compound **5** gradually decreases and the Stokes’ shift increases until the fluorescence returns to its solution state nature (Fig. 5, Additional file 1: Figure S4). This experiment demonstrated that compound **5** binds to the structured protein and not to a denatured protein. In other words, it reports the progressive loss of binding sites on its receptor when the receptor is undergoing a massive structural change.

### Drug like properties of compound **5**

The molecular properties of the compound, such as clogP, clogS, polar surface area etc. (Bickerton et al. 2012) are listed in the Table 3. The clogP value of a compound is the logarithm of its partition coefficient between n-octanol and water. It is a well established measure of the compound’s lipophilicity, which influences its behaviour in a range of biological processes such as solubility, membrane permeability, lack of selectivity and non-specific toxicity (Alam et al. 2011). It has been shown for compounds to have a reasonable probability of being well absorbed, their logP value must not be greater than 5.0 (Lipinski et al. 1997). Besides, the aqueous solubility of a compound is also defined by logS, which significantly affects its absorption and distribution characteristics. Typically, a low solubility goes along with a bad absorption. Most of the drugs on the market have an estimated logS value of about −4. Table 3 lists the polar surface area of the compound as well, which should be less than 140 Å^2^ for a drug molecule (Lipinski et al. 1997). Apart from lipophilicity/solubility and the polar surface area, the molecular weight and the number of hydrogen bond acceptor/donor in compound **5** also follow the Lipinski’s rule of five (Lipinski et al. 1997) to be a candidate drug molecule.

**Table 3:** Molecular properties of compound **5**

Molecular properties	Values
logP^a^	4.56 ± 0.62
logS	−5.97
Polar surface area	94.17 Å^2^
Lipinski’s rule of five	Yes

^a^The data represent mean ± SD

### Molecular modeling

**Fig. 6:**
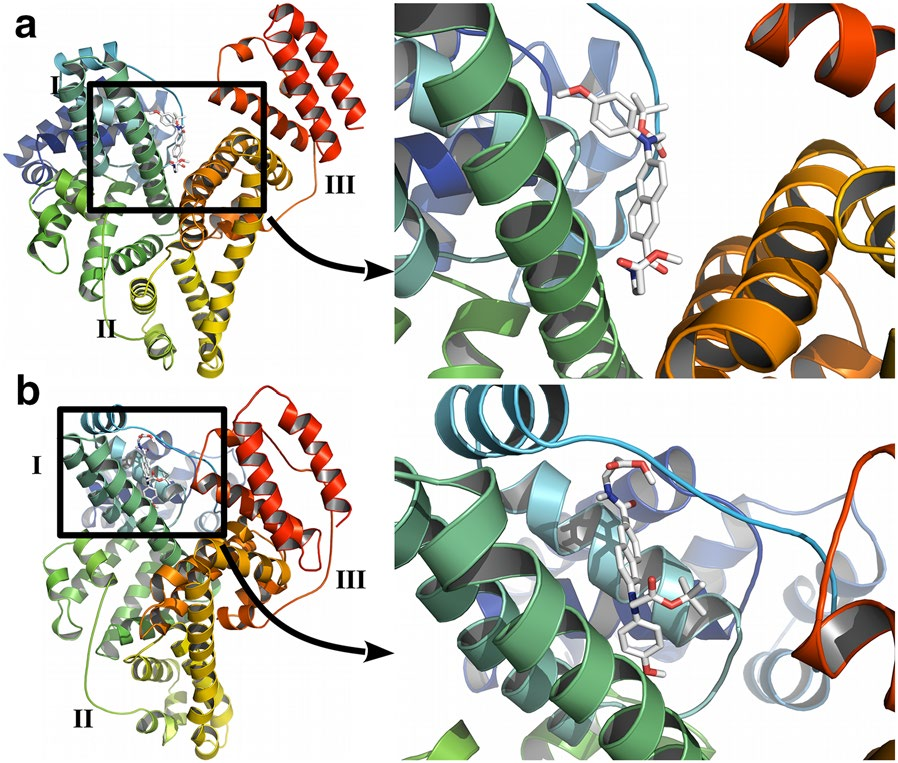
Interaction of compound **5** with serum albumins as obtained from molecular docking experiments. **a** Best binding conformation of compound **5** with BSA and the close up view. **b** Best binding conformations of compound **5** with HSA; it is also shown in close up. AutoDock Vina generated complexes are depicted here. Proteins are shown in *ribbon diagram* and the ligands in stick model. The three domains of serum albumin are marked with *I*–*III*. *Standard color* representation is used to denote the elements, H, N and O in the ligand

In silico molecular docking calculation shows that the interactions of the compound with serum albumins are thermodynamically favorable (Table 4). The binding free energies computed by AutoDock Vina and SwissDock are very similar to that of the experimentally obtained values (Table 2). Molecular docking also provides the insight into the most favorable binding site for these compounds on the serum albumins. The lowest energy complexes obtained by the three different algorithms consistently showed that the binding sites for compound **5** lay in the groove between domain I and domain III of BSA, whereas it was within the domain I in case of HSA (Fig. 6, Additional file 1: Figure S5). This may, in part, explain the enthalpy driven nature of binding with HSA and the entropy driven binding with BSA (Table 2). Moreover, the non-specific nature of the binding is apparent from the lack of clustering in the AutoDock 4.2 results (Additional file 1: Figure S6). We have shown in earlier works that the low energy high frequency clusters in the AutoDock 4.2 output signifies specificity in the binding interactions (Alam et al. 2012; Rudra et al. 2012; Bhowmik et al. 2013). Serum albumin with its many hydrophobic binding pockets acts like a universal receptor for almost all drug molecules. Binding to serum albumin is generally non-specific in nature and driven by mainly hydrophobic interactions, which is evident in the molecular docking results as well (Additional file 1: Figures S7, S8).

**Table 4:** Theoretical binding free energies as obtained by molecular docking experiments using three different algorithms, AutoDock 4.2, AutoDock Vina and SwissDock

Protein	AutoDock 4.2 (kJ mol^−1^)^a^	AutoDock Vina (kJ mol^−1^)	SwissDock (kJ mol^−1^)
BSA	−17.08 ± 0.35	−33.05	−36.15
HSA	−20.78 ± 0.44	−35.56	−35.52

^a^The data represent mean ± SEM

**Fig. 7:**
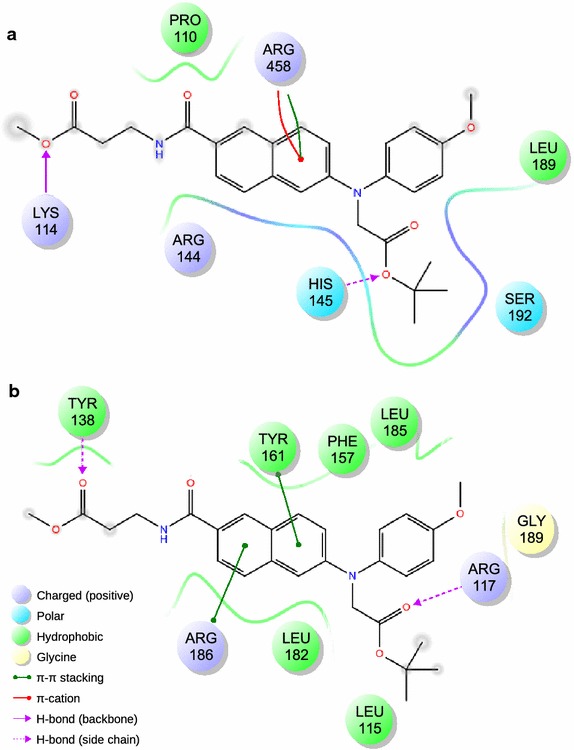
Detailed interaction diagram of compound **5** with serum albumins as obtained by molecular docking experiments. Only the residues common in all three lowest energy complexes generated by AutoDock 4.2, AutoDock Vina and SwissDock are shown. **a** Interacting residues of BSA and the types of interactions with compound **5**. **b** Interacting residues of HSA and the types of interactions with compound **5**

Detailed interaction diagrams of the protein–ligand complexes showing the interacting residues and the types of interactions obtained by three different docking programs (AutoDock 4.2, AutoDock Vina and SwissDock) are given in Additional file 1 (Figures S7, S8). The consensus of the interacting residues of BSA and HSA with compound **5** are produced from those interacting diagrams depicted in Additional file 1: Figures S7, S8 and is shown in Fig. 7. We have found that Lys114 and His145 of BSA forms H-Bond with compound **5**, whereas, Arg458 forms pi-cation interaction. Other important interacting residues of BSA are Arg144, Ser192, Pro110 and Leu189. In case of HSA, it is found that Arg117 and Tyr138 forms H-bond, whereas, Arg186 and Tyr161 forms pi-stacking with compound **5**. Other important interacting residues of HSA are Leu115, Phe157, Leu182, Leu185 and Gly189.

## Conclusion

We have reported here, the spectroscopic behavior and binding parameters of a novel synthetic fluorophore in aqueous buffer and in the presence of albumin proteins. The compound showed drug like properties and bound to serum albumin with the binding constants in low micromolar concentration range. Moreover, the compound showed some interesting properties that could be used to probe the microenvironment, which reflect the immediate surroundings of the molecule inside the target proteins. The compound binding to the hydrophobic sites of serum albumin significantly increased its fluorescence quantum yield, caused significant decrease in the Stokes’ shift indicating changes of excited state geometry of the molecule inside protein binding pocket. We observed an overall effect of the microenvironment of the binding site on the fluorophore. Whether it was hydrophobicity alone or some other factor such as polarity or charge or a combination of all and how those environmental variables correlate with the fluorescence property of the molecule requires further elaborate experiment to understand. In the future experiment we plan to study the fluorescence properties of this molecule in different solution condition, micellar and liposomal or membranous environments.

## Additional file

**Additional file 1.** Detailed procedure of compound **5** synthesis and the characteristic data. ^1^H and ^13^C NMR spectra of compound **5** are given in Additional file. It also contains Figures S1–S8.
