# Supplementary material for: Binding interaction of a novel fluorophore with serum albumins: steady state fluorescence perturbation and molecular modeling analysis
Source: Springerplus. 2015 Sep 24;4:548. doi: 10.1186/s40064-015-1333-8 (PMC4582037; doi:10.1186/s40064-015-1333-8)
Supplement: Supplementary file 1 — Additional file 1. Detailed procedure of compound 5 synthesis and the characteristic data. 1H and 13C NMR spectra of compound 5 are given in Additional file. It also contains Figures S1–S8. [file 40064_2015_1333_MOESM1_ESM.doc]

Supporting Information for

**Binding Interaction of a Novel Fluorophore with Serum Albumins: Steady State Fluorescence Perturbation and Molecular Modeling Analysis**

Uttam Pal^a, 1^, Sumit Kumar Pramanik^b, 1^, Baisali Bhattacharya^a^, Biswadip Banerji^b^, Nakul Chandra Maiti^a^, *

^a^ *Structural Biology & Bioinformatics Division, Council of Scientific & Industrial Research (CSIR)-Indian Institute of Chemical Biology (IICB), Kolkata, West Bengal, India*

^b^ *Chemistry Division, Council of Scientific & Industrial Research (CSIR)-Indian Institute of Chemical Biology (IICB), Kolkata, West Bengal, India*

* Corresponding author. Tel.: +91 33 2499 5940; fax: +91 33 2473 5197.

E-mail address: ncmaiti@iicb.res.in (N.C. Maiti).

^1^ These authors contributed equally to this work.

**Synthesis of Compound 5**


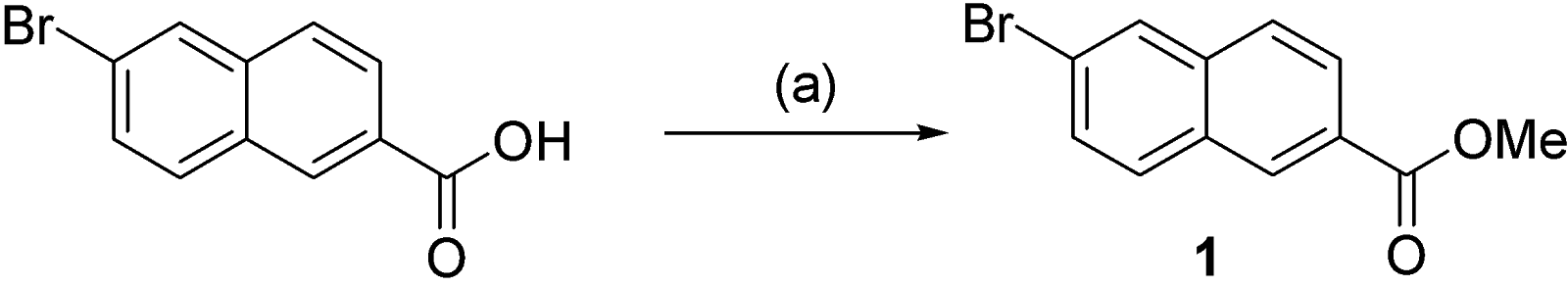


**Scheme S1** Reagent and conditions: (a) Conc. H_2_SO_4_, 0 ^o^C to rt, 6 hrs

To a stirred solution of 6-bromo-2-napthoic acid (1 equiv.) in methanol was added H_2_SO_4_ (10 % by mass) at 0 ^o^C. The reaction was then refluxed for 6 hours. The reaction mixture was then neutralized by 1 M NaOH solution and extracted with ethyl acetate. The ethyl acetate was then concentrated to dryness, and the residue was purified by column chromatography (hexane/ ethyl acetate) to afford **1**.


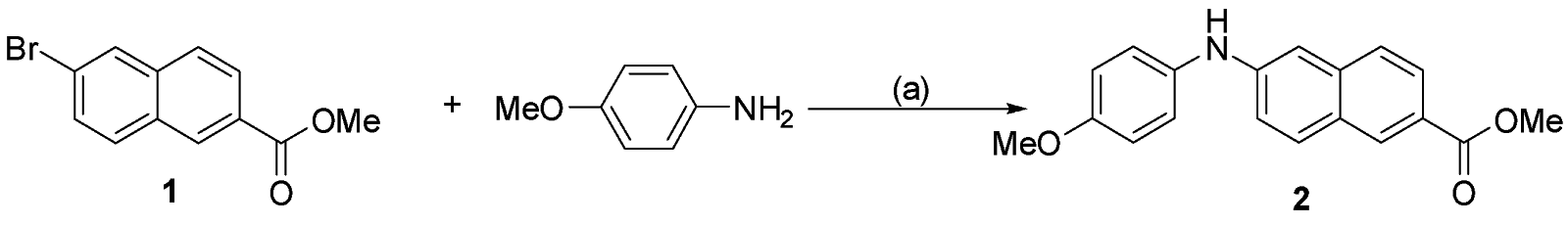


**Scheme S2** Reagent and conditions: (a) palladium (II) acetate (0.05 equiv.), xantphos (0.1 equiv.) and cesium carbonate (3 equiv.), 80 ^o^C, 4 hrs.

To a stirred solution of compound **1** (1 equiv.) and 4-methoxyaniline (1.2 equiv.) in 1,4-dioxane was added palladium(II) acetate (0.05 equiv.), xantphos (0.1 equiv.) and cesium carbonate (3 equiv.). The reaction was then continued at 80 ^o^C for 4 hours. The reaction mixture was filtered, concentrated to dryness, and the residue was purified by column chromatography (hexane/ ethyl acetate) to afford **2**.


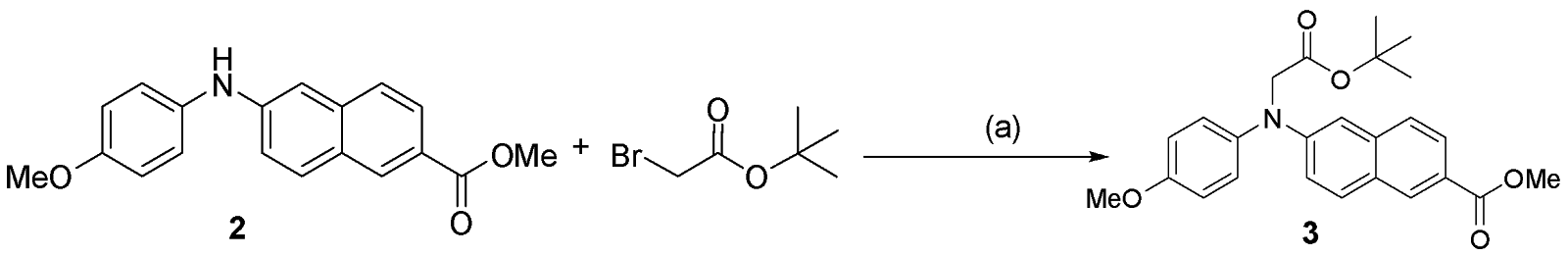


**Scheme S3** Reagent and conditions: (a) potassium tert-butoxide (1.2 equiv.), DMF, 0 ^o^C to rt, 12 hrs.

To a stirred solution of compound **2** (1 equiv.) in dimethyl formamide was added tert-butyl bromo acetate (1.2 equiv.) and potassium tert-butoxide (1.2 equiv.) at 0 ^o^C. The reaction was then continued at room temperature for 12 hours. The reaction mixture was worked up with ethyl acetate and water. The ethyl acetate was concentrated to dryness, and the residue was purified by column chromatography (hexane/ ethyl acetate) to afford **3**.


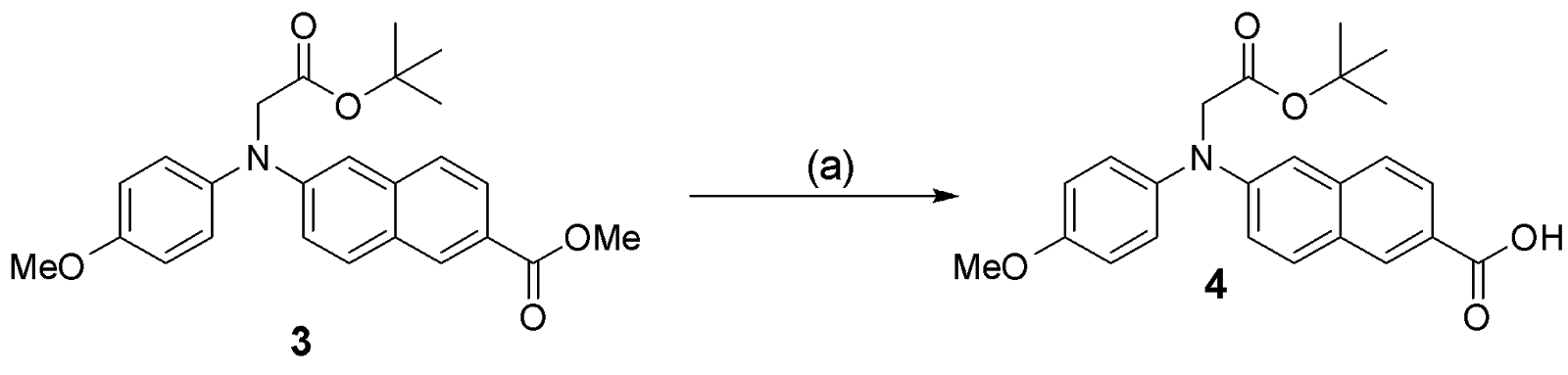


**Scheme S4** Reagent and conditions: (a) Lithium hydroxide (3 equiv.), MeOH-water (5:1), r.t., 2 hrs.

To a stirred solution of compound **3** (1 equiv.) in MeOH-water (5:1) was added lithium hydroxide (3.0 equiv.) at 0 ^o^C. The reaction was then continued at room temperature for 1.5 hours. MeOH was then evaporated and the solution was then neutralized by using 1 M HCl solution and the compound was then extracted with ethyl acetate. The ethyl acetate was then concentrated to dryness to afford **4**, which was carried out to the next step without further purification.


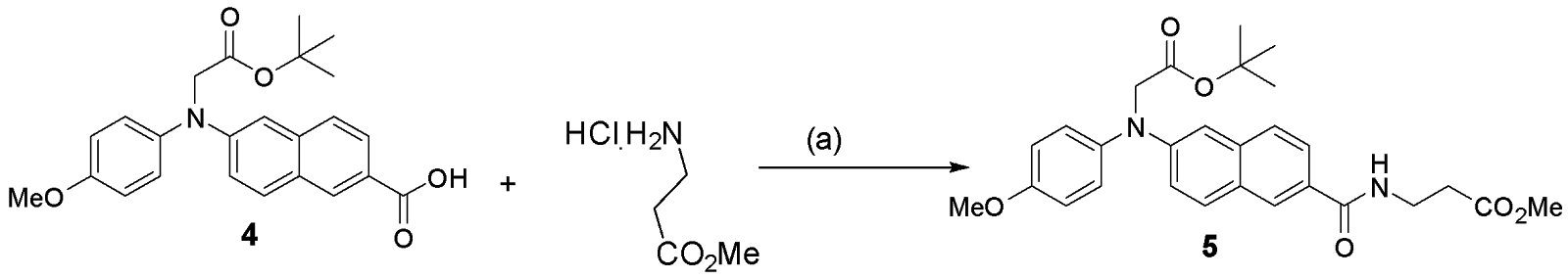


**Scheme S5** Reagent and conditions: (a) EDC.HCl (1.5 equiv.), HOBT (1.2 equiv.), TEA (3 equiv.), 0 ^o^C to rt, 1.5 hrs.

To a stirred solution of compound **4** (1 equiv.) and methyl 3-aminopropanoate hydrochloride (1.2 equiv.) in dry THF was added EDC.HCl (1.5 equiv.), HOBT (1.2 equiv.) and triethyl amine (3 equiv.) at 0 ^o^C. The reaction was then continued at room temperature for 7 hours. The reaction mixture was concentrated to dryness, and the residue was purified by column chromatography (hexane/ ethyl acetate) to afford **5**.

**Characteristic Data**

Methyl 3-(6-((2-(tert-butoxy)-2-oxoethyl)(4-methoxyphenyl)amino)-2-naphthamido)propanoate:

Light yellow solid, m.p.= 214 - 215°C, 1H NMR (600 MHz, CDCl3): δ (in ppm) 1.44 (9 H, s), 2.68 (2 H, t, J = 6.0), 3.72 (3 H, s), 3.76-3.75 (2 H, m), 3.83 (3 H, s), 4.37 (2 H, s), 6.85 (1 H, d, J = 2.4), 6.96-6.94 (4 H, m), 7.27-7.26 (2 H, m), 7.57 (1 H, d, J = 8.4), 7.65 (1 H, d, J = 9.0), 7.70 (1 H, d, J= 6.0), 8.12 (1 H, s); 13C NMR (150 MHz, CDCl3): δ (in ppm) 28.00, 31.87, 33.80, 35.28, 51.79, 55.43, 81.91, 107.26, 114.96, 118.09, 123.95, 126.38, 126.48, 127.26, 127.77, 128.27, 129.65, 136.39, 139.57, 144.55, 157.51, 167.61, 169.77, 173.81; HRMS (FAB+): (M+H) / z Calcd for C28H33N2O6 (M+H)+ 493.2339, Found: (m+H) / z 493.2336.


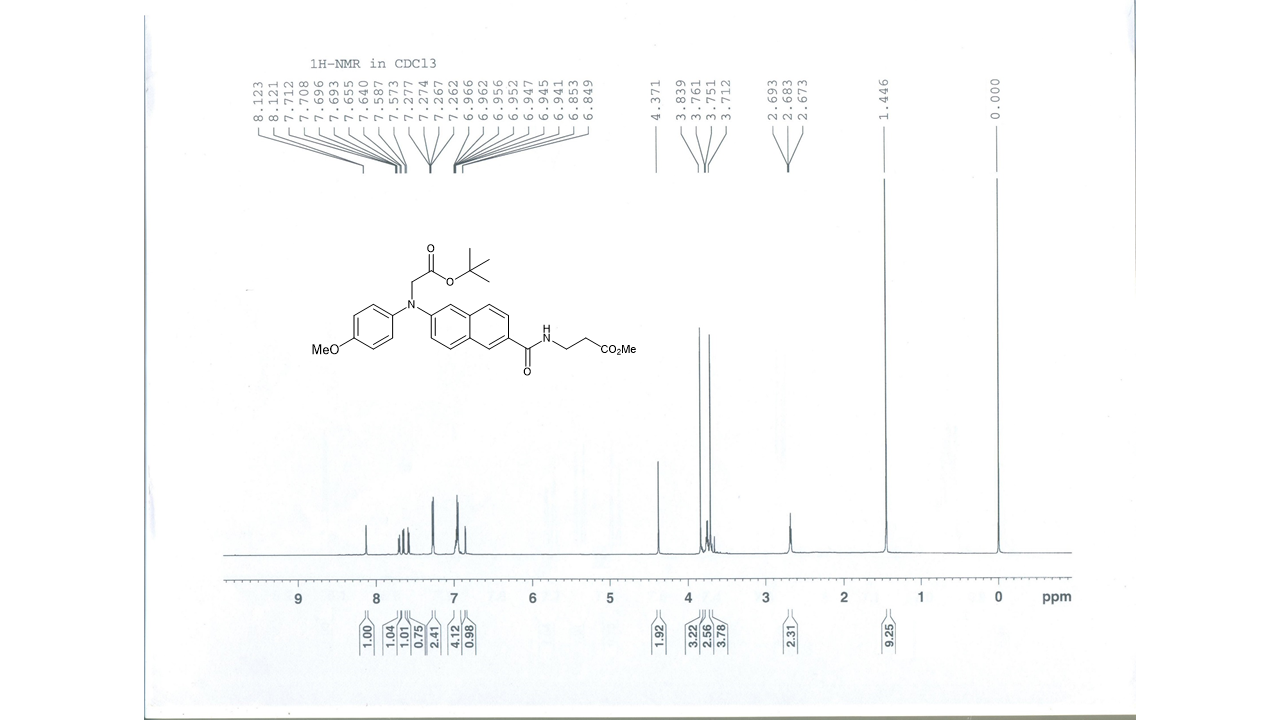


^1^H NMR spectra of compound **5** in CDCl3


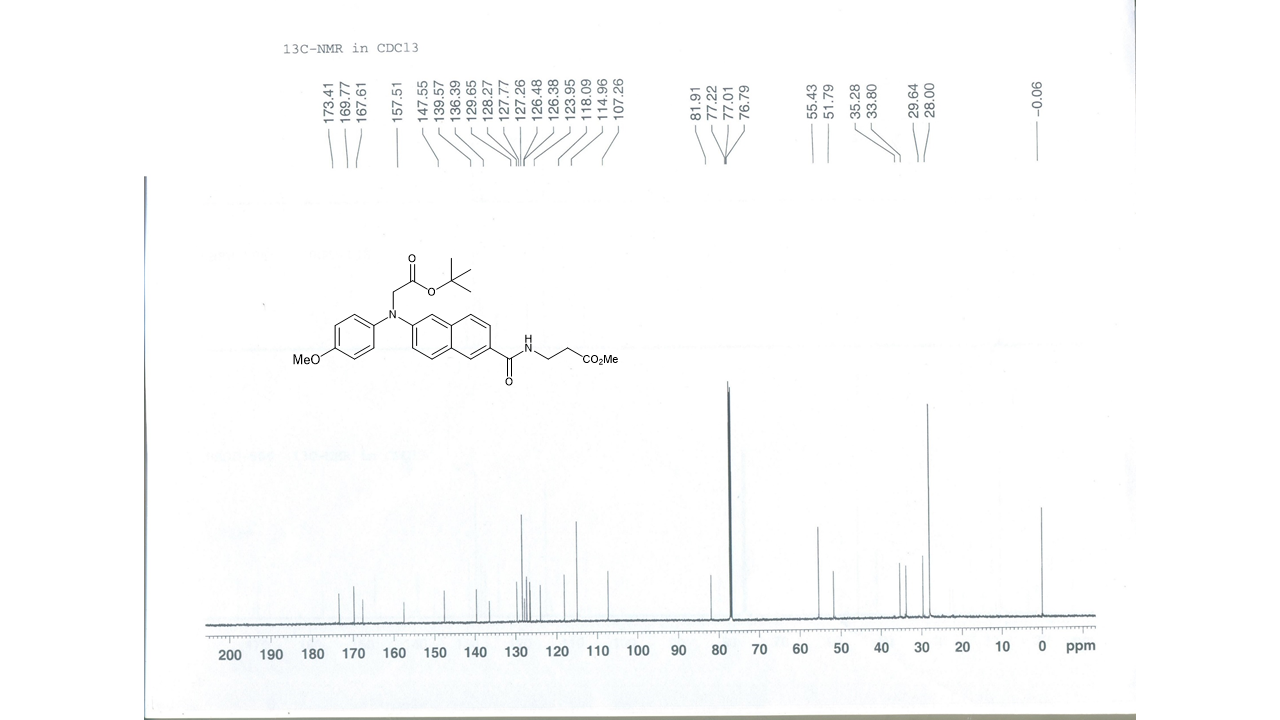


^13^C NMR spectra of compound **5** in CDCl3


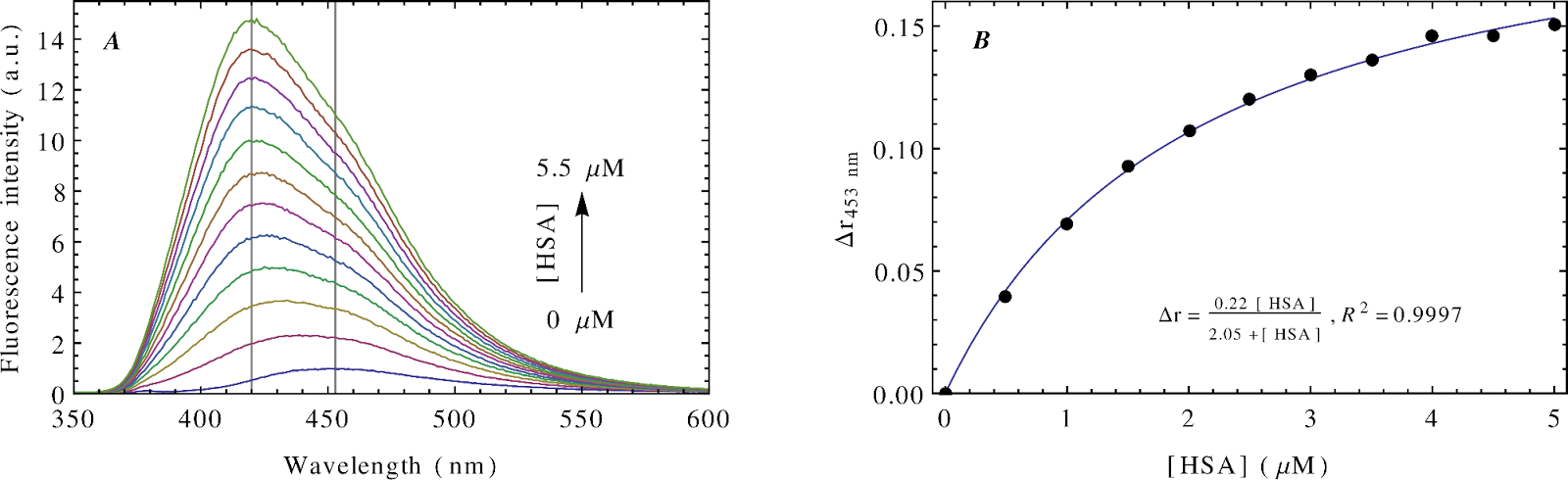


**Figure S1** Fluorescence emission and anisotropy change of compound **5** in presence of serum albumin. Compound **5** concentration was kept constant at 0.5 μM and the protein concentration was varied from 0 through 5.5 μM. (A) Change in the fluorescence emission spectra of compound **5** as a function of HSA concentration. (B) Change in the compound **5** fluorescence anisotropy with increasing concentration of HSA and the fitted Langmuir isotherm.


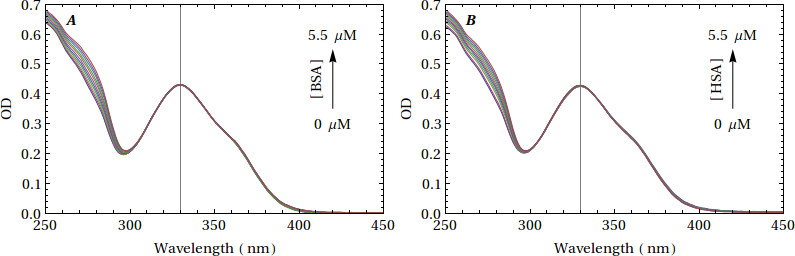


**Figure S2** Absorption spectra of compound **5** as a function of serum albumins. Compound **5** concentration was kept constant at 0.5 μM and the protein concentration was varied from 0 through 5.5 μM. (A) compound **5** absorption spectra with increasing concentration of BSA. (B) compound **5** absorption spectra with increasing concentration of HSA.


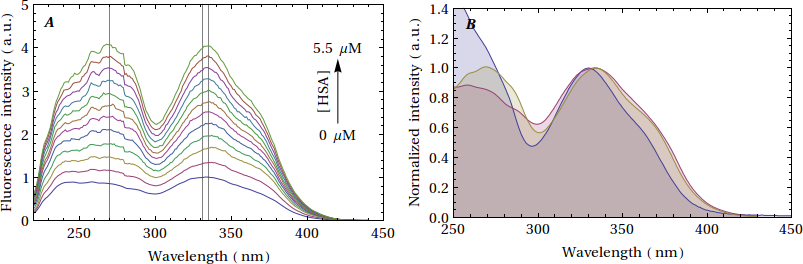


**Figure S3** Compound **5** binding is an excited state phenomenon. (A) Change in the compound **5** fluorescence excitation spectra upon addition of HSA. (B) Normalized absorption (light blue) and fluorescence excitation spectra of compound **5** (light red) overlapped with compound **5** fluorescence excitation spectra (light yellow) in presence of HSA.


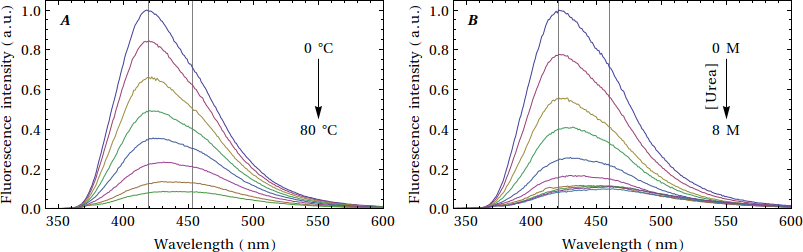


**Figure S4** Compound **5** interaction with denaturing HSA. (A) Change in the fluorescence of HSA compound **5** complex with the increasing temperature. (B) Change in the fluorescence of HSA compound **5** complex with the increasing concentration of Urea.


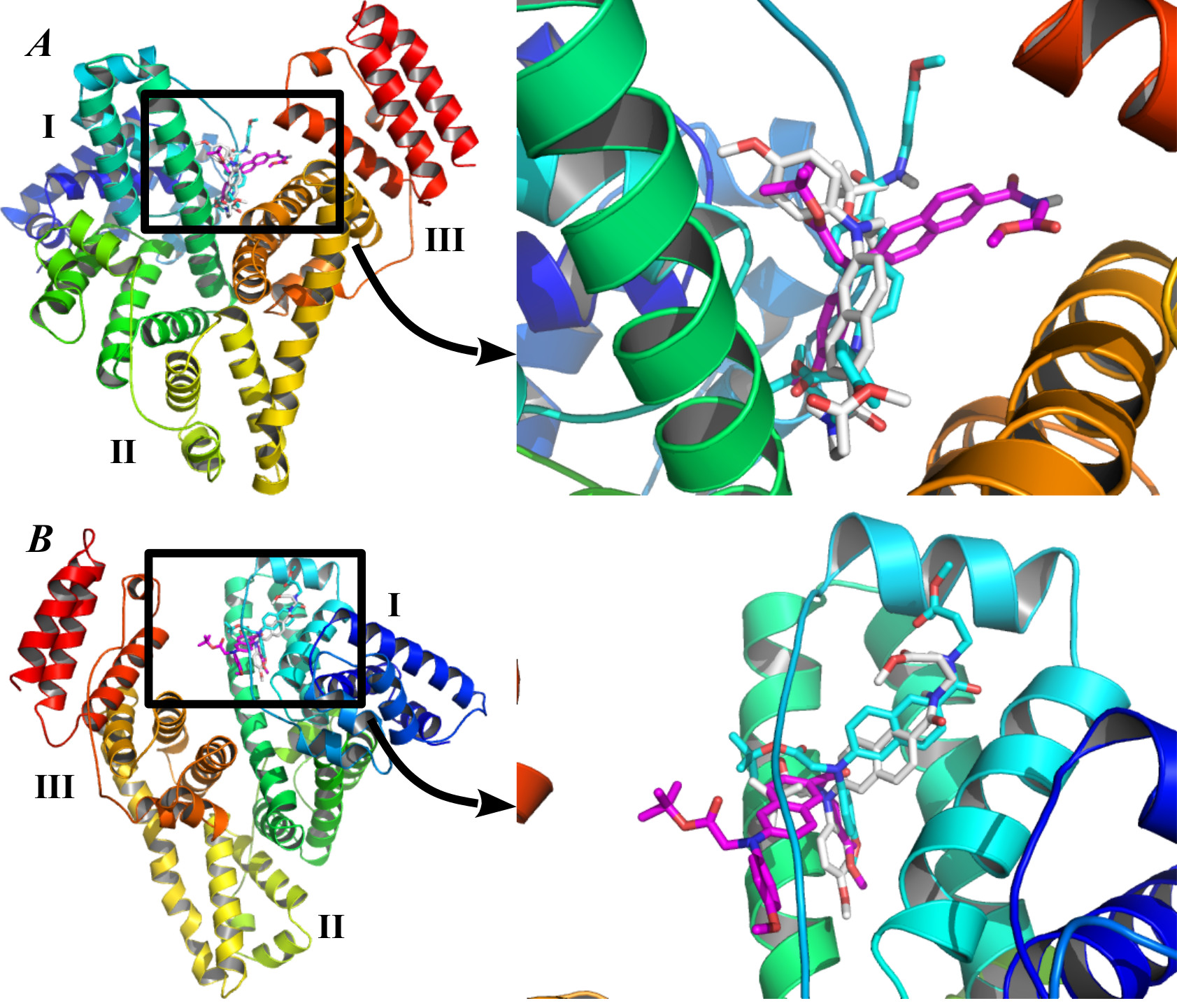


**Figure S5** Interaction of compound 5 with serum albumins as obtained by three different molecular docking algorithms. AutoDock 4.2, AutoDock Vina and SwissDock results are painted in green, white and cyan, respectively. (A) Best binding conformation of compound **5** with BSA and the close up view. (B) Best binding conformations of compound **5** with HSA; it is also shown in close up. Proteins are shown in ribbon diagram and the ligands in stick model. The three domains of serum albumin are marked with I—III. Standard color representation is used to denote the elements, H, N and O in the ligand.

***A***


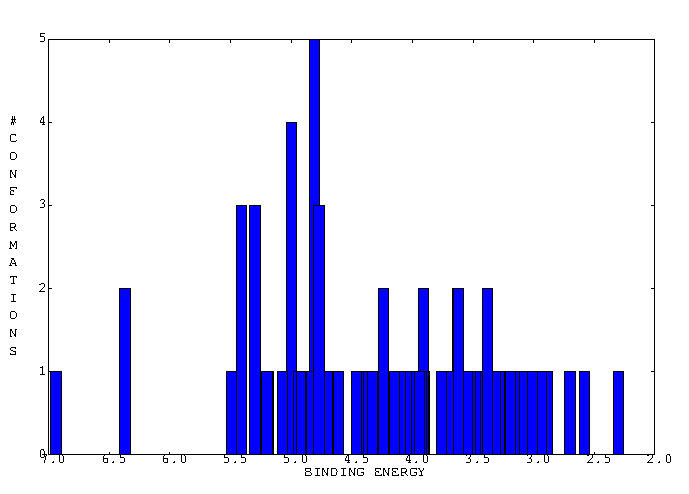


***B***


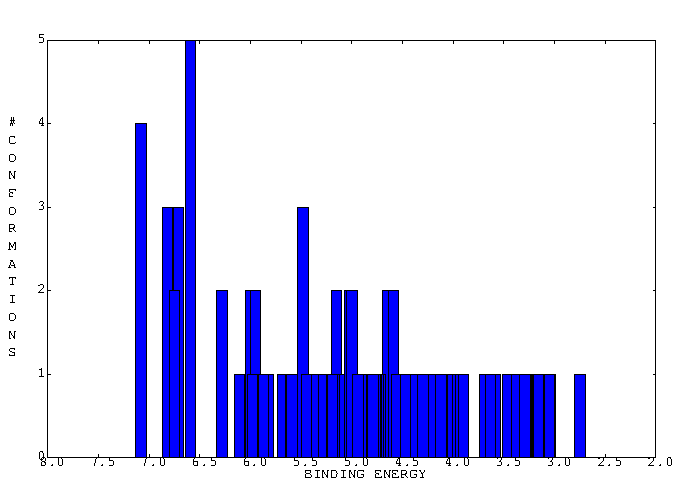


**Figure S6** Cluster frequency distribution of bound conformations obtained from AutoDock 4.2. All the best conformers within 2 Å standard deviation and 0.5 kcal mol^-1^ energy deviations were grouped together. (A) Cluster of BSA bound conformers. (B) Cluster of HSA bound conformers. Binding energy values are negative.

A


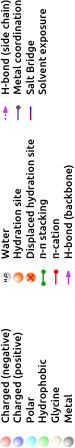

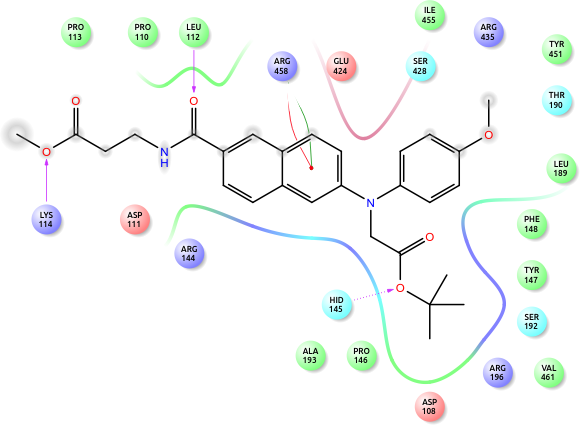


***B***


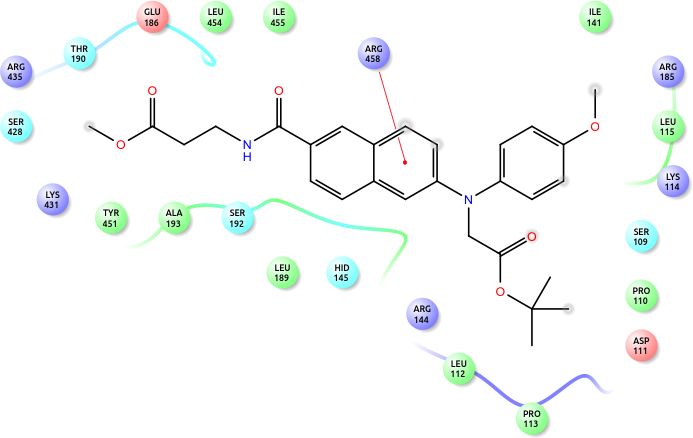


***C***


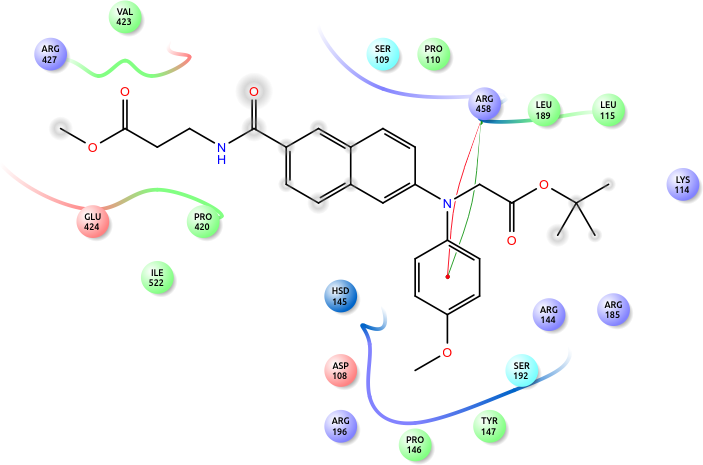


**Figure S7** Interacting residues of BSA with the compound **5** as obtained by molecular docking experiments. (A) Interacting residues and the types of interaction with BSA as obtained by AutoDock 4.2. (B) Interacting residues and the types of interaction with BSA as obtained by AutoDock Vina. (C) Interacting residues and the types of interaction with BSA as obtained by SwissDock. Color codes and the symbolic expressions for different kind of interactions are mentioned.

A


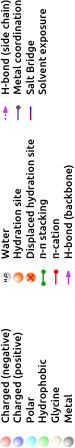

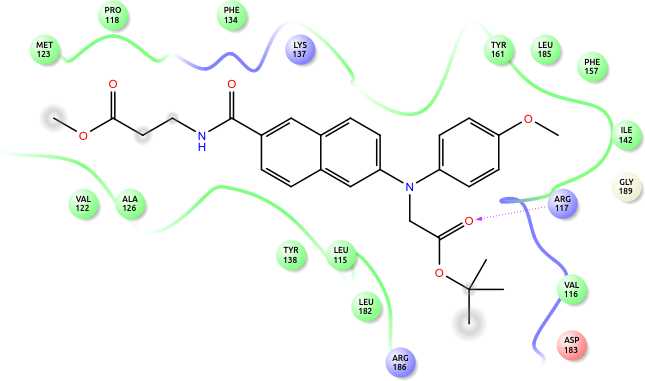


***B***


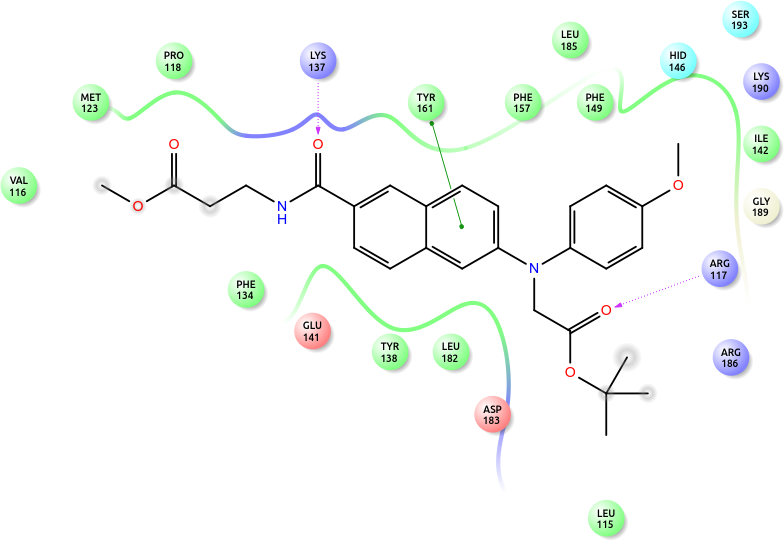


***C***


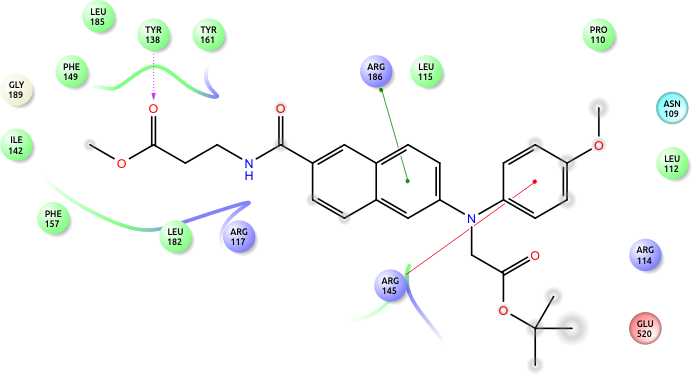


**Figure S8** Interacting residues of HSA with the compound **5** as obtained by molecular docking experiments. (A) Interacting residues and the types of interaction with HSA as obtained by AutoDock 4.2. (B) Interacting residues and the types of interaction with HSA as obtained by AutoDock Vina. (C) Interacting residues and the types of interaction with HSA as obtained by SwissDock. Color codes and the symbolic expressions for different kind of interactions are mentioned.
